# Properties of Additively Manufactured Electric Steel Powder Cores with Increased Si Content

**DOI:** 10.3390/ma14061489

**Published:** 2021-03-18

**Authors:** Giulia Stornelli, Antonio Faba, Andrea Di Schino, Paolo Folgarait, Maria Rita Ridolfi, Ermanno Cardelli, Roberto Montanari

**Affiliations:** 1 Dipartimento di Ingegneria Industriale, Università degli Studi di Roma “Tor Vergata”, Via del Politecnico 1, 00133 Roma, Italy; giulia.stornelli@students.uniroma2.eu (G.S.); roberto.montanari@uniroma2.it (R.M.); 2 Dipartimento di Ingegneria, Università degli Studi di Perugia, Via G. Duranti, 06125 Perugia, Italy; antonio.faba@unipg.it (A.F.); ermanno.cardelli@unipg.it (E.C.); 3 Seamthesis Srl, Via IV Novembre 156, 29122 Piacenza, Italy; paolo.folgarait@seamthesis.com (P.F.); mariarita.ridolfi@seamthesis.com (M.R.R.)

**Keywords:** additive manufacturing, FeSi steels, magnetic properties

## Abstract

In this paper, the best laser powder bed fusion (L-PBF) printing conditions for FeSi steels with two different Si content (3.0% and 6.5%) are defined. Results show very strict processing window parameters, following a lack of fusion porosity at low specific energy values and keyhole porosity in correspondence with high specific energy values. The obtained microstructure consists of grains with epitaxial growth starting from the grains already solidified in the underling layer. This allows the continuous growth of the columnar grains, directed parallel to the built direction of the component. The magnetic behaviour of FeSi6.5 samples, although the performances found do not still fully reach those of the best commercial electrical steels (used to manufacture magnetic cores of electrical machines and other similar magnetic components), appears to be quite promising. An improvement of the printing process to obtain thin sheets with increased Si content, less than 0.5 mm thick, with accurate geometry and robust structures, can result to an interesting technology for specific application where complex geometries and sophisticated shapes are required, avoiding mechanical machining processes for electrical steel with high silicon content.

## 1. Introduction

The huge diffusion of electrical devices and systems has led to realize ever more performing and reliable electrical machines such as electric motors, electric generators, electric transformers, and inductive filters. The ability to develop equipment, in terms of energy conversion, economic convenience, and durability, is depending on several aspects, among them the magnetic materials used [1,2,3,4].

The continuous and considerable increment of the use of the switching power supplies, and the increment of their working frequency, makes the magnetic power losses of electrical machines and filters crucial for the improvement of the efficiency of the system [1,2,3,4]. Magnetic losses, also called core losses when referring to magnetic cores, are mainly due to the application of time-varying magnetic fields to the magnetic material. The change of the magnetization state of the material dissipates energy which is transferred to the surrounding environment in the form of heat [5]. The choice of the magnetic material, the design and the optimization of the magnetic core geometry, and the related manufacturing techniques are the fundamental issues to get the best energy performance [6]. More generally, there are different types of magnetic losses, but the most significant contribution is due to the hysteresis and eddy current losses. The first occurs when a cyclic magnetic field is applied, also in quasi-static conditions, and not all the energy of the magnetic field is returned to the circuit when the field is removed. These depend on the grain size, texture, and lattice defects that provide pinning sites on the magnetic domain walls while eddy current losses are associated with the conductive nature of the magnetic core. The application of magnetic field leads to induced loop currents that generate additional energy losses over than hysteresis losses [5,7,8]. Moreover, in the case of low electrical resistivity or large component thickness, the induced currents prevent the complete penetration of the magnetic field into the core. This limits the complete magnetization of the component, thus the conversion efficiency of the electric machines.

Silicon steels represent one of the most important classes of soft magnetic materials used in magnetic applications [9]. Within this, the FeSi steels with Si content between 2 wt.% and 7 wt.%, together with an appropriate electrical resistivity [10], guarantee excellent electromagnetic proprieties [11]: for this reason, they are widely used in the ferromagnetic cores of electrical motors, generators, electrical transformers, etc. [12].

The commonly adopted process to manufacture ferromagnetic cores consists of stacking thin sheets of FeSi steel, coated with a dielectric material [1]. The strategy based on the use of 0.2–0.5 mm thick and coated sheets allows for interrupting the induced currents’ circulation path and reduces eddy current losses [13,14]. Based on this approach, good magnetic properties are conferred to the components. At the same time, this approach provides technological limits.

It is well known that FeSi steel with 6.5 wt.% Si offers the best soft magnetic proprieties [15,16] such as: high magnetic saturation, low magneto-crystalline anisotropy, low magnetostriction, and, above all, high electrical resistivity [12,17,18]. However, commercially, steels with Si content below 3.5–4.0 wt.% are usually preferred [19]. High silicon steels (Si content ≥4.5%) are intrinsically brittle to such an extent that their poor workability does not allow the production of thin sheets through the rolling process [16,20].

The brittleness of the high Si steels is due to the tendency to form phases with ordered structures during cooling [16,21,22]. The FeSi steel has a body-centered cubic (bcc) cell [15]; during the solidification, the material undergoes a rearrangement of the Si atoms in the Fe matrix, forming two types of ordered structures, B2 and DO3 [23,24,25]. It is known that the dislocations movement within an ordered structure forms antiphase boundaries [26,27] with a consequent increase in hardness and of brittleness [26]. This strongly reduces the ductility of the material and limits the workability at low temperatures [12]. Moreover, the formability of FeSi alloys also depends on the control of the twinning and therefore on the speed of the plastic deformation process [28].

Owing to the intrinsic brittleness of FeSi steels, some alternative techniques have been developed for manufacturing components which cannot be processed through the conventional rolling process (e.g., [29,30,31]). Many efforts have been focused on the production of thin sheets in FeSi magnetic steel with high Si content through different methods such as rapid quenching [32,33] to prevent the order-disordered phase transition or chemical vapor deposition (CVD) [15,16,34] in which Si is added by thermal diffusion to low Si content sheets. Other techniques include direct powder rolling [29], strip casting [30], physics vapor deposition (PVD) [31], or spray forming [24,35,36]. All of these techniques proved to be expensive, industrially challenging and of limited practical application. Several papers report about sintered soft magnetic cores (e.g., [11,37,38]) with insulating materials between alloy particles (such as SiO_2_ [37] and Al_2_O_3_ [38]). Such insulators act in reducing the generation of inter-particle eddy current. 

For several years, the Additive Manufacturing (AM) has been imposing itself as an established technology for the manufacture of metal materials on an industrial scale [39,40]. Among these, the Fe-based alloys are of particular importance [41,42]. Recently, it has been shown that AM represents a valid technological alternative for the production of magnetic FeSi steels with high Si content [9]. Currently, the most widespread AM technology, in industrial applications, is the Powder Bed Fusion (PBF). The PBF technology mainly uses the laser source as an energy source, an alternative being the electron source. The components are built by melting metal powders, layer by layer [43], directly from Computer Aided Design (CAD) models [44,45]. The high cooling rates involved in the laser melting process allow for avoiding the typical disordered-ordered phase transition in FeSi steels. Moreover, through the AM process, it is possible to manufacture components with complex geometry [43] in order to optimize the geometry of ferromagnetic cores independently from the mechanical properties of the steel or the limits of conventional technologies.

Up to now, the scientific research on the production of magnetic materials and components through Additive Manufacturing technology is quite young [13]; however, the possibility of producing complex and more performing components represents a powerful technology drive to innovate the production of ferromagnetic cores and meet the challenging requests from the electric traction sector and, in general, from electric propulsion transport [46,47]. The contributions present in the literature refer to the experimental manufacture of ferromagnetic cores in FeSi steel with Si content between 6.7 wt.% and 6.9 wt.% [9,10,12,13,48] and are focused on setting the parameters of the melting process (e.g., laser power and scan speed) to minimize the porosity and produce components with high magnetic properties. Some strategies [13] concern the building of ferromagnetic cores having internal geometry with slits or the alternation of materials of different nature. In this way, the presence of air inside the slits or a low conductivity material emulates the behavior of the dielectric materials typically used in conventional production.

The scope of the present research project is to use the Direct Melting Laser Sintering (DMLS) technology to produce ferromagnetic cores with high Si content with high performances, providing a competitive alternative for industrial applications. In particular, this work reports the results of a feasibility study for the production of FeSi steels through Direct Melting Laser Sintering (DMLS) technology. Two FeSi steels have been considered with Si content of 3.0 and 6.5 wt.% and the metallurgical characteristics in relation to the process technological parameters. The magnetic behavior has been studied on different geometries of the transverse section of the samples. For both steels, three different types of sections have been examined to suitably optimize the effect of the geometry on the mitigation of the eddy current losses.

## 2. Materials and Methods

Two FeSi steels with Si content of 3.0 and 6.5 wt.% have been investigated. The powders were produced by gas–atomization and their chemical compositions (wt.%) are reported in Table 1.

**Figure 1:**
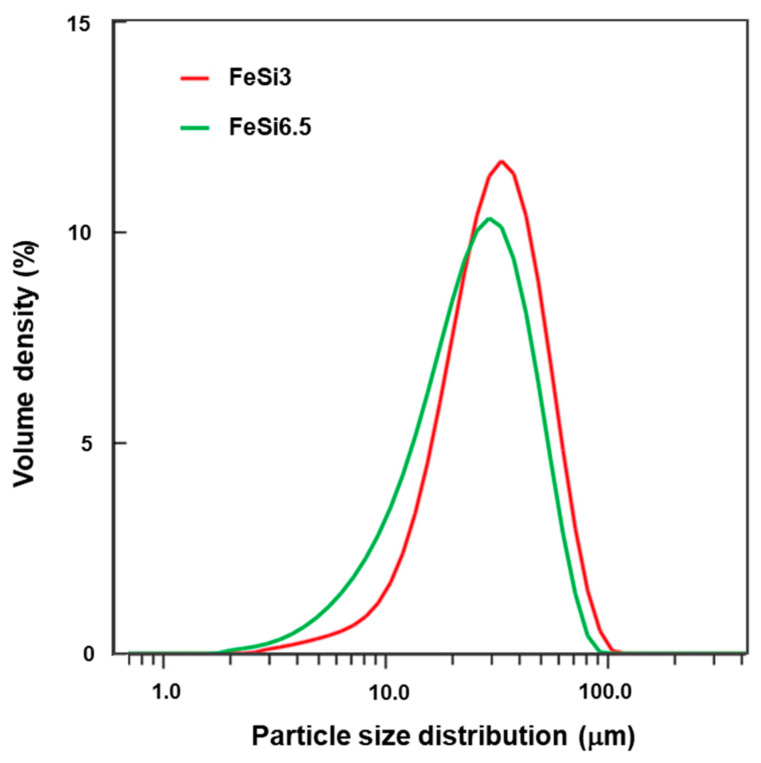
Particle size distribution of the as-received powders of FeSi3 and FeSi6.5 steels, used in the L-PBF system.

Particle size distributions (PSD) (Figure 1), measured by laser diffraction methods (Mastersizer 3000, Malvern-Panalytical, Malvern, UK), show average sizes of about 31 μm and 25 μm for FeSi3 and FeSi6.5 steel powders, respectively.

**Figure 2:**
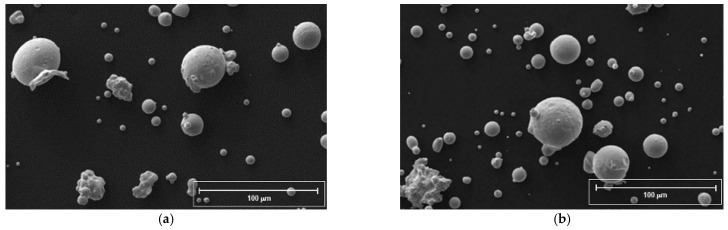
Powder steel morphology of FeSi3 (**a**) and FeSi6.5 (**b**), SEM images.

Some important parameters of PSD are shown in Table 2. The morphology of powders was generally spherical with some satellites (Figure 2) and with an ellipticity value of the particles, for both steels, close to 1.

The powders were processed through a system with L-PBF technology to produce test samples. The fabrication was carried out in an EOS M290 machine (EOS Gmbh, Offenbach am Main, Germany) equipped with an Yb fiber laser with a nominal diameter of 100 μm and a Gaussian power distribution curve. The platform temperature was kept at 200 °C, the maximum temperature allowed by the device, to reduce as far as possible the residual stresses induced by thermal gradients during the process. The process was carried out under an Argon atmosphere with oxygen content below 0.4%.

At first, 20 (twenty) small cubes (11 mm × 11 mm × 11 mm) of both steels were fabricated to analyze the effect of process parameters on the density. Such preliminary work identified the window of the process parameter suitable to manufacture the samples for electromagnetic properties’ measurement.

**Figure 3:**
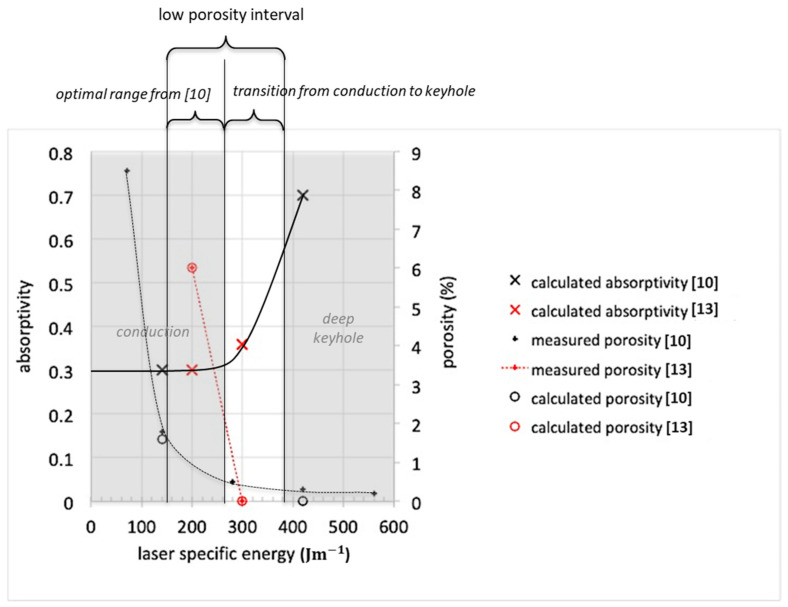
Absorptivity, printing modes’ limits (conduction and keyhole), and porosity, derived through numerical modeling of the processes described in [10,13], referring to steels with 6.7% and 6.9% Si.

A first estimate of optimal printing parameters has been achieved by applying a proprietary simulation model developed for the L-PBF process [49,50,51], to printing processes reported in the literature [10,13]. The model describes the interaction, on a microscopic scale, between metal powder, laser source, and gas mixture, within the process chamber. The behavior of incident laser energy absorbed by the metal (laser absorptivity) and porosity, as a function of specific laser energy, is reported in Figure 3. Two modes are put in evidence: *’conduction’* and *’keyhole’* modes: the first one is characterized by almost flat liquid pool free surface and small penetration, whereas the second one realizes at higher laser specific energies. This second mode provokes the onset of metal boiling and increases the presence of deeper cavities (keyhole) and pool penetration [52]. The results shown in Figure 3 identify the range of specific laser energy: 150–400 Jm^−1^ as optimal since porosities due to both lack of fusion and keyhole should be minimized. Moreover, an indication on the yield of energy above which the incidence of cracks becomes unacceptable is provided in [10] as 280 Jm^−1^. Based on the calculated absorptivity curve (Figure 3), the abruptly increasing trend of the crack index [10] results in initiating at the metal vaporization onset. Much lower crack incidence is found at conduction printing regimes. The simplified model applied in this work allows for assessing the thermal gradient across the mushy zone, highly responsible for hot cracks opening, only in the conduction regime, showing the large effect of the platform temperature (the calculated thermal gradient drops from about 3000 K/mm down to 1500 K/mm, increasing the platform temperature from 200 K to 400 K). On the other hand, the model fails to provide reliable thermal gradient values for the transition and keyhole modes [49], for which the real deformed shape of the gas/liquid interface approaches the vaporization temperature isotherm to the solidification front, thus largely increasing the thermal gradient in the mushy zone.

All of the above considerations lead to concluding that this kind of alloy requires being printed at a low enough laser energy input to avoid transition or keyhole regimes, preventing both porosities and cracks.

On the basis of this first estimate, a list of parameter sets (in terms of v, P and E), to be experimentally investigated, has been compiled (Table 3). The values of scan speed *v* and laser power *P* were varied, respectively, in the ranges 0.5–1 ms^−1^ and 75–240 W.

Moreover, the values of the scan line spacing and the thickness of each powder layer were kept constant at 60 μm and 30 μm, according to [9,13], and the samples were built with a scanning strategy which turned the laser direction 67° at each layer. 

The density of the cubic samples was determined using the Archimedes method (AB54 Mettler Toledo, Columbus, OH, USA). The samples were weighed first in air and then in distilled water in the suspension state as a counter-proof. The measurements were performed three times for each sample, and the relative density was expressed as the average value.

The samples were cut along a plane parallel to the build direction (BD), polished and observed using an optical microscope (Eclipse LV150NL, Nikon, Tokyo, Japan) to evaluate the presence and the nature of porosities and cracks, related to the process parameters.

The same polished surfaces were etched a solution of 2% Nital for 20–40 s to observe the morphology of the melt pool and the solidification microstructure.

Later, samples of specific geometries were manufactured to measure the magnetic proprieties of FeSi3 and FeSi6.5 steels. For this characterization, three ring samples with a square section with side 10 mm, internal diameter of 40 mm, and external diameter of 60 mm were manufactured.

It is known that, in general, the annealing heat treatment temperature is dependent on microstructure in the as-built samples [51]. Anyway, in this class of materials, it has been reported that the best annealing conditions are temperature of 1150 °C for 1 hour [48]. Therefore, ring samples were heat treated accordingly.

Since texture plays a crucial role on the magnetic behavior of FeSi alloys, flat samples prepared by using the same process parameters chosen for manufacturing the ring samples have been examined by X-ray diffraction (XRD) (PW 1729, Philips, Eindhoven, The Netherlands). XRD measurement on flat samples was carried out on the surface parallel to the built direction. The analyzed surface is parallel to the radial direction of ring samples used for magnetic measurements with crystallographic texture parallel to the applied magnetic field. XRD spectra have been collected by using the Mo-Kα radiation (λ = 0.15408 nm) in the 2Θ angular range 15–55 degrees (step scanning mode with steps of 0.05 degrees and counting time per step of 5 s). The texture has been evaluated from the comparison of the peak intensities of each sample with those of an Fe sample with randomly oriented grains taken from the JCPDS-X-ray database-File 6-696 [53].

**Figure 4:**
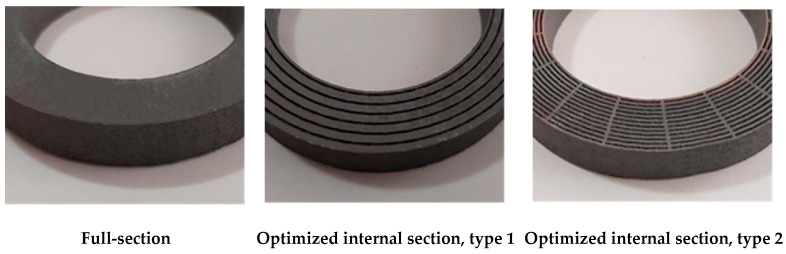
Internal section of sample-ring manufactured for magnetic characterization of FeSi3 and FeSi6.5.

Furthermore, three different geometries of the ring cross section were tested. In particular, for both steels, one ring sample with a full-section and two ring samples with optimized internal geometry (type 1 and type 2) were manufactured. The printed rings are shown in Figure 4.

The objective of testing optimized geometries with internal slits is to use the atmosphere filling them as a dielectric material, in order to reduce eddy current losses.

The samples just described were manufactured using the process parameters (*E*, *v*, and *P*) that allowed the maximum densification of cubic samples. In particular, the best process parameters for FeSi3 ring samples were *E* = 250 Jm^−1^, *v* = 1 ms^−1^, *P* = 250 W, while for FeSi6.5 were *E* = 200 Jm^−1^, *v* = 0.835 ms^−1^, *P* = 167 W.

**Figure 5:**
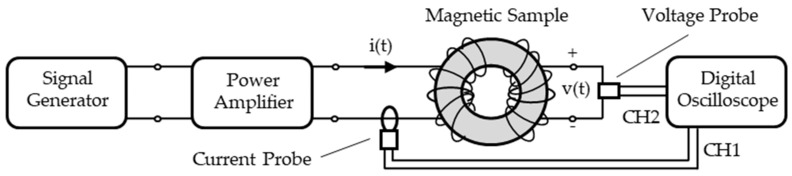
Experimental set-up for the magnetic property measurement of the samples in FeSi3 and FeSi6.5.

The magnetic properties, measured for the six samples described above, were obtained by means of the experimental set-up shown in Figure 5.

These tests were performed to measure the dynamic hysteresis loops, the anhysteretic curves and the power losses, from the virgin state up to the saturation region of the magnetic samples. The test method is based on the standard volt-ampere procedure, largely used and described in literature [54,55]. The magnetic field and the magnetic induction, inside the material, are determined by means of the measurement of the exciting current and the induced voltage on a couple of suitable coils wrapped on the test sample. This method results in being very reliable and useful for the detection of several properties of the magnetic materials [56,57,58,59]. The measuring equipment used for the tests are listed in Table 4.

The signal generator and the power amplifier generate an exciting alternate current i (t) on the primary coil; that current is measured and stored by the current probe and channel 1 of the digital oscilloscope (CH1). At the same time, the induced voltage v(t), on the secondary coil, is measured and stored by the voltage probe and the channel 2 of the digital oscilloscope (CH2).

The ring-sample is magnetized with different magnetic induction values changing the values of the amplitude of the exiting alternate current. The magnetic field H(t) and the magnetic induction B(t) are determined by the following equations, as required by the standard volt-ampere method implemented here [54,55]:

(1) $$H\left(t\right)=\;\frac{N\;i\left(t\right)}{l}$$

(2) $$B\left(t\right)=\;\frac{1}{N\;S}\;\int v\left(t\right)\;dt$$

*N* is the number of the coils turns, *l* is the average length of the sample-ring, and *S* is the cross-section area of the sample-ring.

The graphical plot of the H and B sampling data draws the hysteresis loops of the sample-ring, the hysteresis loop vertexes draw the anhysteretic curves, and the area of the hysteresis loops divided by the exciting current period gives the power losses [54,55].

## 3. Results and Discussion

### 3.1. Metallurgical Features of FeSi Steel Samples Manufactured through L-PBF Technology

Preliminary tests were performed to establish the process parameters suitable for the production of magnetic components and the relative density values of the cubic samples are reported in Table 5 and Table 6, respectively, for FeSi3 and FeSi6.5 steels. They showed that, in order to reduce the internal porosity fraction, the best combination of process parameters for the FeSi3 steel was: *E* = 250 Jm^−1^, *v* = 1 ms^−1^, *P* = 250 W. The S7 sample of FeSi3, manufactured with these process parameters, was found to have a relative density of 99.99%. The best sample of FeSi6.5 steel, S3, also returned a relative density of 99.99% using the following process parameters: *E* = 200 Jm^−1^, *v* = 0.835 ms^−1^, *P* = 167 W.

For the best process condition, in terms of relative density, the fabrication of additional cubic samples was performed in order to verify the repeatability of the condition of the manufacturing process. For the FeSi3 and FeSi6.5 samples made from the second production, the relative density values were the same as the samples produced in the preliminary manufacturing step.

**Figure 6:**
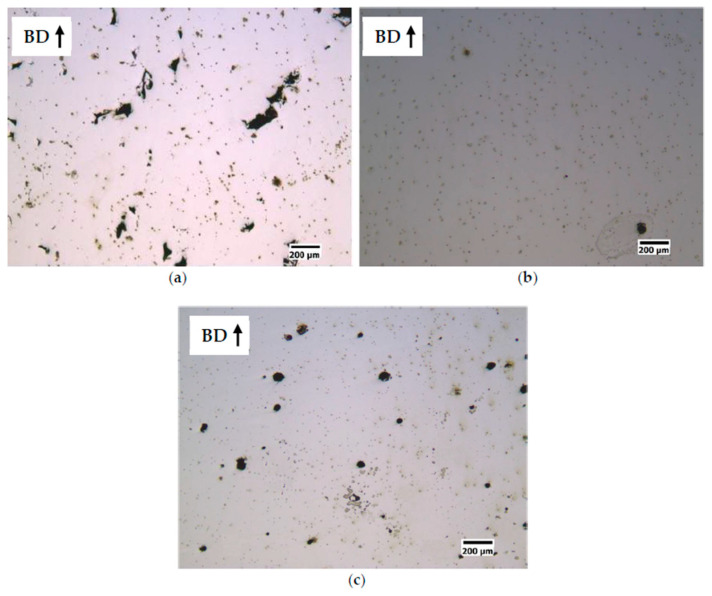
Effect of the specific laser energy E [Jm^-1^] on the densification of FeSi3 steel samples. (**a**) sample S1 (E = 150 Jm^−1^, v = 0.5 ms^−1^, P = 75 W), relative density of the sample 99.93% and pores with irregular shape; (**b**) sample S7 (E = 250 Jm^−1^, v = 1 ms^−1^, P = 250 W), relative density of the sample 99.99%; (**c**) sample S18 (E = 350 Jm^−1^, v = 0.5 ms^−1^, P = 175 W), relative density of the sample 99.98% and pores with spherical shape.

**Figure 7:**
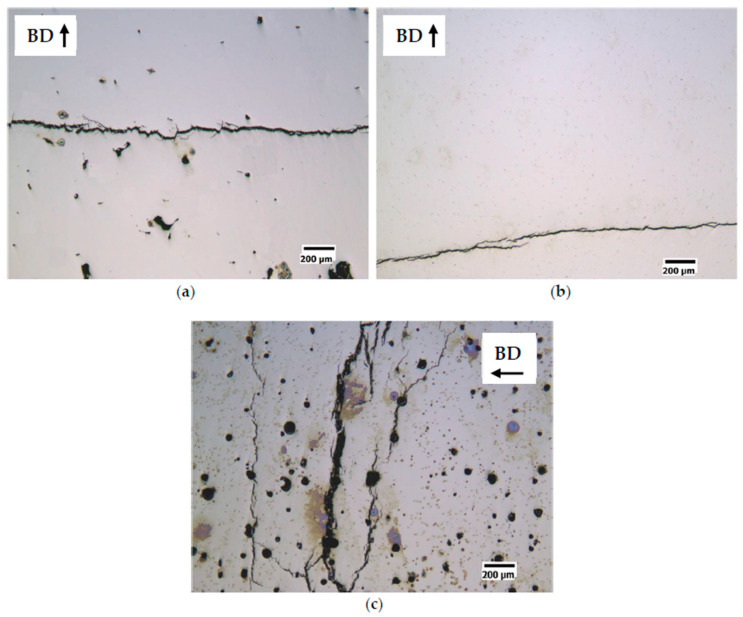
Effect of the specific laser energy *E* (Jm^−1^) on the densification of FeSi6.5 steel samples. (**a**) sample S1 (*E* = 150 Jm^−1^, *v* = 0.5 ms^−1^, *P* = 75 W), relative density of the sample 99.93% and pores with irregular shape; (**b**) sample S3 (*E* = 200 Jm^−1^, *v* = 0.835 ms^−1^, *P* = 167 W), relative density of the sample 99.99%; (**c**) sample S18 (*E* = 350 Jm^−1^, *v* = 0.5 ms^−1^, *P* = 175 W), relative density of the sample 99.98% and pores with spherical shape.

From a first observation of the polished sections (Figure 6 and Figure 7) of the cubic samples, the FeSi3 steel is completely crack-free, whereas all the samples of FeSi6.5 steel exhibit cracks and the presence of these defects becomes more relevant as the specific laser energy increases.

The process parameters related to the S3-FeSi6.5 sample can be considered as the best compromise in terms of sample densification and cracks formation.

Micrographic analysis of porosity showed that low specific laser energy *E* (Jm^−1^) results in the formation of irregularly shaped pores (Figure 6a and Figure 7a for S1-FeSi3 and S1-FeSi6.5 samples, respectively). This particular shape can be associated with empty spaces among not perfectly melted powder particles. The relative density of the S1 sample was the lowest among all the samples for both steels, 99.93% and 99.96%, respectively for FeSi3 and FeSi6.5. Conversely, with a high specific laser energy *E* [Jm^−1^], the pores assume a spherical shape (Figure 6c and Figure 7c for S18-FeSi3 and S18-FeSi6.5 samples, respectively).

**Figure 8:**
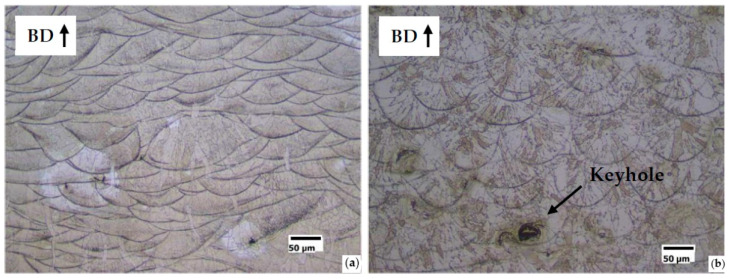
Example of transition from conduction melting to keyhole formation. (**a**) optical micrograph of the longitudinal section to the build direction (BD) of S1-FeSi6.5 sample (*E* = 150 Jm^−1^, *v* = 0.5 ms^−1^, *P* = 75 W), conduction melt mode; (**b**) optical micrograph of the longitudinal section to the build direction (BD) of S18-FeSi6.5 sample (*E* = 350 Jm^−1^, *v* = 0.5 ms^−1^, *P* = 175 W), keyhole mode.

The relative density of the S18 samples was 99.98% for both FeSi3 and FeSi6.5 steels. The sphericity of the pores can be associated with the entrapment of metal vapor and partially ionized gas in the liquid pool. The subsequent fast cooling freezes the spherical shape of the final cavity [52]. This type of phenomenon occurs typically when quite deep keyhole cavities are formed in the meltpool, filled with plasma from both the process gas and the metal vapor. In this regard, the transition from conduction to keyhole mode is also testified by the increasing depth of the track transverse section, observable in the sample etched surfaces. Examples are given in Figure 8a for the conduction mode (shallow penetration) and in Figure 8b for the keyhole mode (high penetration).

**Figure 9:**
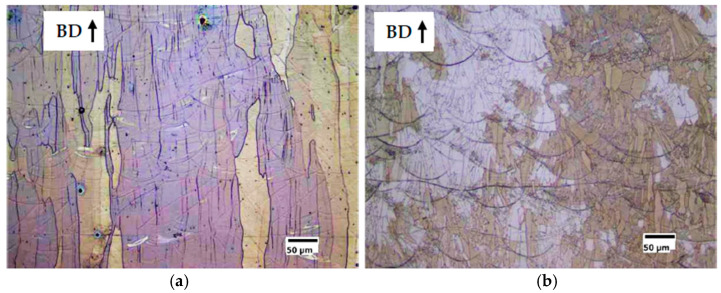
Columnar microstructure of longitudinal sections along the built direction (BD) of the highest density samples: (**a**) S7-FeSi3 (*E* = 250 Jm^−1^, *v* = 1 ms^−1^, *P* = 250 W) and (**b**) S3-FeSi6.5 (*E* = 200 Jm^−1^, *v* = 0.835 ms^−1^, *P* = 167 W).

The solidification microstructures are fully columnar as shown in Figure 9a,b, respectively for the samples S7-FeSi3 and S3-FeSi3, showing the highest density. This microstructure is expected for FeSi steels produced by AM [9] because, on each new layer, the solidification grains grow epitaxially from the consolidated material of the underling layer. This allows the continuous growth of the columnar grains, directed parallel to the built direction of the component.

**Figure 10:**
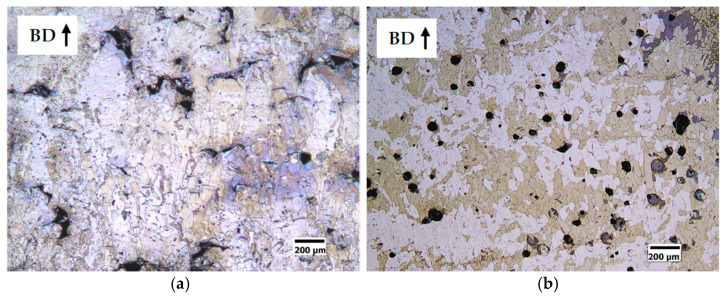
Example of non-columnar microstructure in the high porosity samples. (**a**) S1-FeSi3 (*E* = 150 Jm^−1^, *v* = 0.5 ms^−1^, *P* = 75 W) and (**b**) S20-FeSi6.5 (*E* = 400 Jm^−1^, *v* = 0.6 ms^−1^, *P* = 240 W).

A relevant result is that the fully columnar microstructure is lost in the high porosity samples. In case of a lack of fusion, the interruption of the columnar growth is caused by the not complete interconnection among the tracks (Figure 10a). In case of the keyhole, the alteration of thermal and fluid-dynamic conditions near the solidification front could favor the nucleation of new grains (Figure 10b).

**Figure 11:**
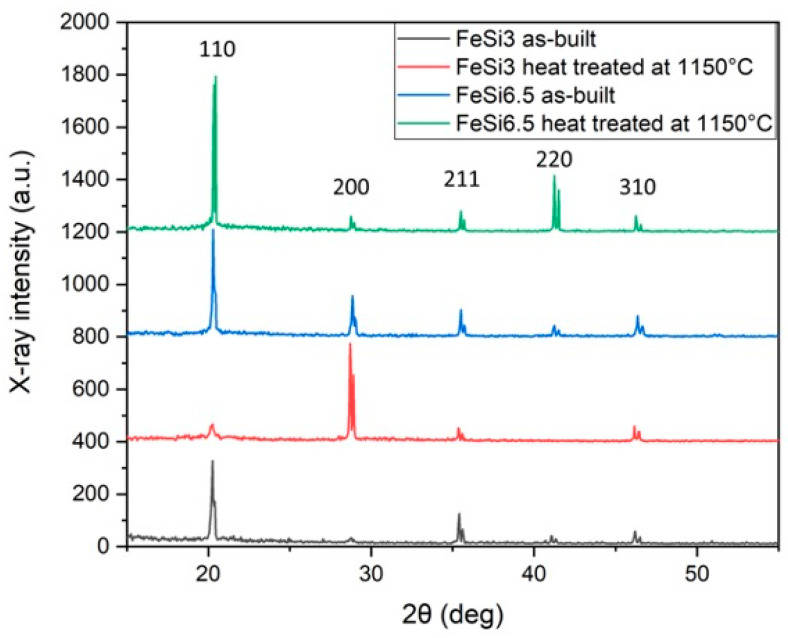
XRD spectra of FeSi3 and FeSi6.5 steels samples, in as-built condition and after heat treatment at 1150 °C for one hour.

XRD measurements have been carried out to evaluate the texture originating from the process parameters used for manufacturing the rings. The XRD spectra of FeSi3 and FeSi6.5 steels samples, in as-built condition and after heat treatment at 1150 °C for one hour, are shown in Figure 11.

The intensities of XRD peaks have been determined and normalized to that of the strongest reflection *(I* = 100) of each sample (Table 7).

The comparison of the normalized intensities with those of a Fe sample with randomly oriented grains [53] allowed for evaluating the texture.

The FeSi3 alloy in as-built condition exhibits a strong <211> texture because the intensity of {211} peak is ~2.3 times that corresponding to a material with random grain orientation. Such texture evolves into the <001> cubic texture after the heat treatment at 1150 °C. This is good for magnetic applications because the hard magnetization <111> direction is not present in the vertical and horizontal planes of the sample.

In the case of as-built FeSi6.5 alloy, the texture is cubic and becomes <110> Goss after heating. The possible mechanisms underlaying the texture evolution following heat treatment may be found in the some heterogenous properties in the as-built sample (e.g., elongated grains, plastic strain due to the thermal stress, etc.). To better understand the mechanisms leading to a texture change following heat treatment at 1150 °C, further analyses are underway.

### 3.2. Magnetic Behavor of FeSi Steel Samples Manufactured Through L-PBF Technology

**Figure 12:**
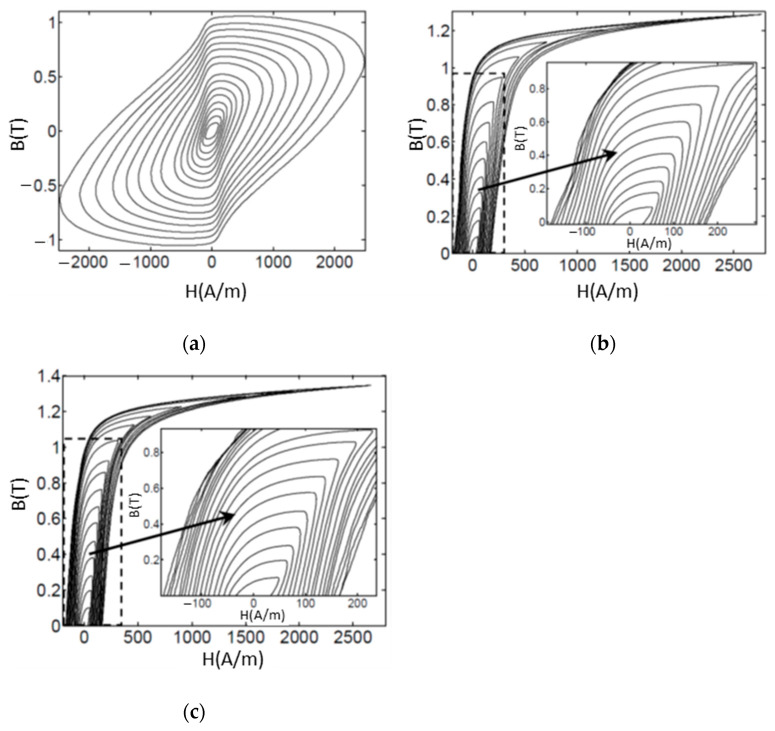
Dynamic hysteresis loops at 50 Hz, samples FeSi3, (**a**) sample with full-section; (**b**) sample with optimized Section 1; (**c**) sample with optimized Section 2.

**Figure 13:**
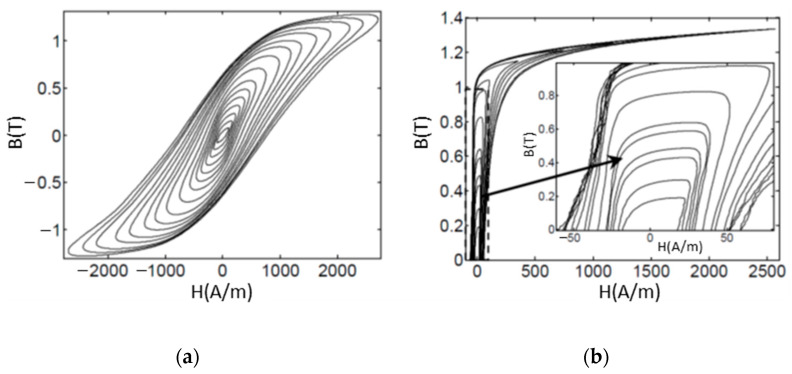

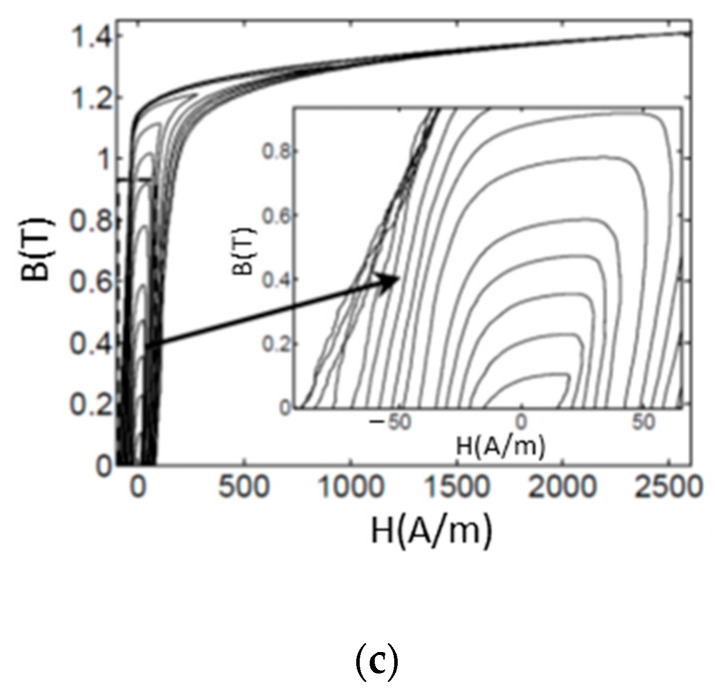
Dynamic hysteresis loops at 50 Hz, samples FeSi6.5, (**a**) sample with full-section; (**b**) sample with optimized Section 1; (**c**) sample with optimized Section 2.

The dynamic hysteresis loops of the FeSi steel samples, using exciting currents with frequency f = 50 Hz, are presented in Figure 12 and Figure 13.

**Figure 14:**
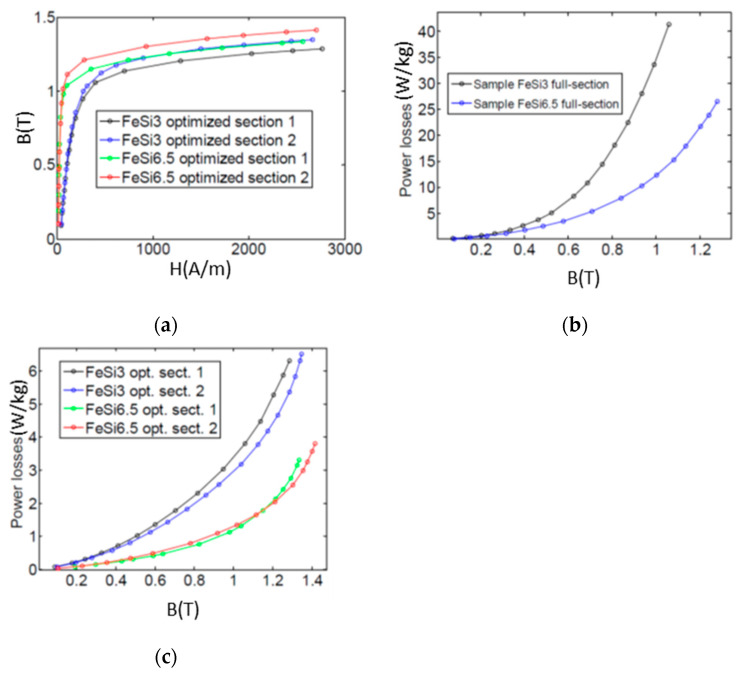
(**a**) Anhysteretic curves of the samples with optimized sections; (**b**) power losses of the samples with full-section; (**c**) power losses of the samples with optimized sections.

The eddy current effects are clearly visible in the full-section samples where the areas of the loops are very large because of the high power losses. These effects result in being strongly mitigated in case of the samples with the optimized sections. The FeSi6.5 samples show less eddy current effects in comparison with the FeSi3 samples, this fact resulting in the higher magnetization capability (see Figure 14a) and reduced power losses over 50% (see Figure 14b,c). The sample in FeSi6.5 with optimized section type 2 shows the best performances, although still far from the commercial FeSi laminated cores; for instance, the power losses result in being about two times higher in comparison with commercial cores of non-oriented FeSi 3% steel made of sheets of 0.35 mm thickness [52].

The poor performances of the printed cores, compared to the conventional Si steels, could originate from bridges left among the metallic lamellae by entrapped powder particles, which heavily compromise the expected insulation. Moreover, although the presence of cracks in the ring samples is highly reduced compared to more massive components, they still represent a source of performance decay. Finally, the thickness of steel sheets adopted in conventional magnetic cores is thinner than those additively manufactured in this work, allowing a higher reduction of eddy currents.

## 4. Conclusions

An experimental campaign was carried out to investigate the possibility of fabricating ferromagnetic cores of improved performances by means of the L-PBF technique using an EOS M290 system (EOS Gbmh, Offenbach am Main, Germany) and FeSi steels with 3.0 and 6.5 wt.% Si content.

The main critical issue to get high density and crack free printed parts is the very narrow range of process parameters: the porosities are caused by a lack of fusion at low laser energies and keyhole onset at high energies while the cracks are due to the high thermal gradients.

At optimized operating conditions, a relative density close to 1 is obtained for both the tested steels. A fully columnar solidification microstructure along the built direction is observed, due to the epitaxial growth starting from the already consolidated material of the underlying layers.

The FeSi3 alloy in as-built condition exhibits a strong <211> texture that evolves into the <001> cubic one after the heat treatment at 1150 °C. In the case of as-built FeSi6.5 alloy, the texture is cubic and becomes <110> Goss after heating. The resulting preferred grain orientations observed in both alloys represent a good result because the hard magnetization <111> direction is not present in the vertical and horizontal planes of the samples.

The samples prepared and analyzed in this paper show a promising magnetic behavior. In particular, the FeSi6.5 steel appears to be quite promising as it shows less eddy current effects in comparison to FeSi3 resulting in higher magnetization capability and reduced power losses over than 50%.

An improvement of the printing process can result in an interesting and promising technology for specific application where complex geometries and sophisticated shapes of the magnetic core are required, avoiding mechanical machining processes for electrical steel with high silicon content. To this scope, further steps forward can be envisaged in:

(1) the fabrication of very thin sheets, less than 0.5 mm;

(2) the reduction of thermal stresses responsible for cracks by increasing the platform temperature; 

(3) post-processing of the components in order to eliminate contact points among the steel sheets.

## Figures and Tables

**Table 1:** Chemical composition (wt.%) of the as-received powders of FeSi3 and FeSi6.5 steels used in the L-PBF system.

Steel	Fe	Si	C	O
**FeSi3**	Bal.	3.0	0.009	0.0001
**FeSi6.5**	Bal.	6.5	0.008	0.0001

**Table 2:** Particle size distribution measured by the laser diffraction method. D_10_, D_50_, and D_90_ mean the particle size at 10 vol%, 50 vol%, and 90 vol%, respectively.

Steel	D_10_ (μm)	D_50_ (μm)	D_90_ (μm)
**FeSi3**	14.4	30.8	56.4
**FeSi6.5**	9.7	25.3	49.0

**Table 3:** Process parameters set (specific laser Energy *E*, scanning speed *v* and laser power *P*) investigated to fabricate the 20 (twenty) cubic samples of both FeSi3 and FeSi6.5 steels.

**Samples**	**S1**	**S2**	**S3**	**S4**	**S5**	**S6**	**S7**	**S8**	**S9**	**S10**
***E* (Jm^−1^)**	150	150	200	200	225	250	250	275	275	275
***v* (ms^−1^)**	0.50	1.00	0.83	0.50	0.75	0.67	1.00	0.50	0.61	0.94
***P* (W)**	75.0	150.0	167.0	100.0	168.8	167.0	250.0	137.5	167.0	259.0
**Samples**	**S11**	**S12**	**S13**	**S14**	**S15**	**S16**	**S17**	**S18**	**S19**	**S20**
***E* (Jm^−1^)**	300	300	300	310	325	325	325	350	350	400
***v* (ms^−1^)**	0.56	0.86	1.00	0.70	0.51	0.80	1.00	0.50	0.74	0.60
***P* (W)**	167.0	259.0	300.0	217.0	167.0	259.0	325.0	175.0	259.0	240.0

**Table 4:** Test equipment used for the magnetic property measurement.

Test Equipment	Model
Signal Generator	HM8130 (Hameg^©^, Mainhausen, Germany)
Power Amplifier	BOP36-5 (Kepco^©^, Naju, South Korea)
Current Probe	RT-ZC03 (Rohde & Schwarz^©^, Munich, Germany)
Voltage Probe	P2220 (Tektronix^©^, Beaverton, OR, USA)
Digital Oscilloscope	SDS5054X (Siglent^©^, Shenzhen, China)

**Table 5:** Relative density % of cubic samples of FeSi3 steel. The density taken as the reference was 7.68 gcm^−3.^

	**S1**	**S2**	**S3**	**S4**	**S5**	**S6**	**S7**	**S8**	**S9**	**S10**
**Relative Density (%)**	99.936	99.995	99.995	99.974	99.996	99.994	99.997	99.969	99.991	99.995
	**S11**	**S12**	**S13**	**S14**	**S15**	**S16**	**S17**	**S18**	**S19**	**S20**
**Relative Density (%)**	99.994	99.995	99.993	99.993	99.986	99.995	99.991	99.986	99.994	99.985

**Table 6:** Relative density % of cubic samples of FeSi6.5 steel. The density taken as the reference was 7.44 gcm^−3.^

	**S1**	**S2**	**S3**	**S4**	**S5**	**S6**	**S7**	**S8**	**S9**	**S10**
**Relative Density (%)**	99.968	99.998	99.998	99.993	99.998	99.997	99.995	99.991	99.996	99.996
	**S11**	**S12**	**S13**	**S14**	**S15**	**S16**	**S17**	**S18**	**S19**	**S20**
**Relative Density (%)**	99.994	99.996	99.994	99.993	99.989	99.996	99.997	99.983	99.994	99.981

**Table 7:** Intensity of the XRD peaks of FeSi3 and FeSi6.5 steels samples, in as-built condition and after heat treatment at 1150 °C for one hour. Peak intensities are normalized to the most intense one of each XRD pattern (I = 100). The intensities of a Fe sample with randomly oriented grains (JCPDS-X-ray database-File 6-696) are reported for estimating the texture of the examined materials.

Steel	Normalized Intensity of {h k l} Peaks					
	{110}	{200}	{211}	{220}	{310}	Texture
**JCPDS (6-696)**	100	20	30	10	12	Random
**FeSi3 as-built**	100	5	70	8	9	<211>
**FeSi3/1h at 1150 °C**	15	100	12	-	16	<001>
**FeSi6.5 As-built**	100	38	25	10	19	<001>
**FeSi6.5/1h at 1150 °C**	100	8	13	37	10	<110>

## Data Availability

The data presented in this study are available on request from the corresponding author.
